# The Potential for Spatial Distribution Indices to Signal Thresholds in Marine Fish Biomass

**DOI:** 10.1371/journal.pone.0120500

**Published:** 2015-03-19

**Authors:** Emilie Reuchlin-Hugenholtz, Nancy L. Shackell, Jeffrey A. Hutchings

**Affiliations:** 1 Department of Biology, Dalhousie University, P.O. Box 15000, Halifax, Nova Scotia, B3H 4R2, Canada; 2 Oceans Science Division, Department of Fisheries and Oceans, Bedford Institute of Oceanography, P.O. Box 1006, Dartmouth, Nova Scotia, B2Y 4A2, Canada; 3 Centre for Ecological and Evolutionary Synthesis, Department of Biosciences, University of Oslo, P.O. Box 1066, Blindern, NO-0316, Oslo, Norway; Aristotle University of Thessaloniki, GREECE

## Abstract

The frequently observed positive relationship between fish population abundance and spatial distribution suggests that changes in distribution can be indicative of trends in abundance. If contractions in spatial distribution precede declines in spawning stock biomass (SSB), spatial distribution reference points could complement the SSB reference points that are commonly used in marine conservation biology and fisheries management. When relevant spatial distribution information is integrated into fisheries management and recovery plans, risks and uncertainties associated with a plan based solely on the SSB criterion would be reduced. To assess the added value of spatial distribution data, we examine the relationship between SSB and four metrics of spatial distribution intended to reflect changes in population range, concentration, and density for 10 demersal populations (9 species) inhabiting the Scotian Shelf, Northwest Atlantic. Our primary purpose is to assess their potential to serve as indices of SSB, using fisheries independent survey data. We find that metrics of density offer the best correlate of spawner biomass. A decline in the frequency of encountering high density areas is associated with, and in a few cases preceded by, rapid declines in SSB in 6 of 10 populations. Density-based indices have considerable potential to serve both as an indicator of SSB and as spatially based reference points in fisheries management.

## Introduction

Fisheries managers establish abundance limit and target reference points (indicator benchmarks) for commercially exploited fishes to ensure the maintenance of healthy and productive populations and enable the recovery of depleted populations. Scientists will often estimate the biomass of the reproductive part of fish populations or spawning stock biomass (SSB) as a measure of abundance, and recommend target and limit reference points for fisheries based on expected outcomes for SSB [1]. To date, however, many overfished populations have not rebuilt at forecasted rates [2,3].

The spatial structure of fish populations is potentially equally as important in maintaining long-term sustainable fish populations as SSB, however spatial indicators are not typically used by fisheries management [4,5]. Total biomass is merely one aspect of the viability of a population and poor viability can be indicated by atypical spatial distributions [6]. The absence of information about spatial structures in fisheries management can increase the probability of overexploitation [7]. When relevant spatial distribution information is integrated into fisheries management and recovery plans, risks and uncertainties associated with a plan based solely on the SSB criterion would be reduced. Hence, complementary indicators that incorporate spatial distribution information have considerable potential for strengthening management and recovery plans [5]. The potential of spatial distribution indicators to signal changes in biomass has been recognized [8] and efforts towards integrating spatial distribution indicators in assessment methods have been made [6,9,10]. We build on those efforts by comparing the functionality of various spatial distribution methods as indicators for SSB in groundfish fisheries management. We then assess the potential utility and development of fisheries management reference points based on spatial criteria.

There is potential for the development of spatial distribution indices, based on the positive relationship between the abundance of populations and their spatial distribution, a pervasive macro-ecological pattern across a wide range of terrestrial [11,12] and marine species [13–15]. Both density dependent and density independent factors limit the spatial distribution of populations and species by altering living conditions and influencing survival, reproduction, vital rates and abundance [16,17]. Consequently, positive abundance-spatial distribution relations have been explained by abundance-environment relationships [18], abundance-occupancy relationships [15], as well as a combination of the two [19].

We examine abundance-occupancy and density patterns across space, which have been explained predominantly by meta-population [20,21] and density dependent habitat selection (DDHS) theory [22,23]. According to the former, a meta-population is partitioned into several interacting but spatially separated subpopulations that occupy distinct areas or patches [20]. Migration between patches decreases the probability of local extinction, thus serving as a ‘rescue effect’ for meta-populations of conservation concern [24]. The rate of immigration per patch increases as the proportion of patches that are occupied increases, and this can generate a positive relationship between local abundance and the number of occupied patches [16]. Ideal free distribution theory suggests that individuals are capable of choosing the most suitable habitat and are free to occupy that habitat, resulting in a population distribution that reflects available resources [22]. Based on the ideal free distribution, DDHS accounts for a positive abundance—spatial distribution relationship resulting from the dispersal of individuals or populations into suboptimal habitats with increases in abundance. This association occurs because, at low abundance, individuals or populations occupy mainly optimal habitat. As abundance increases, competition for resources also increases, eventually leading to a decrease in habitat suitability, in turn resulting in the spread of individuals or populations into suboptimal habitat [13,23,25].

A relationship between abundance and spatial distribution implies that changes in spatial distribution may impact population abundance and eventually persistence [26]. The relationship between range size and extinction risk is generally negative and non-linear, with species of small ranges experiencing disproportionately higher rates of extinction than those with intermediate range sizes [16,27]. A decline in population range size can decrease gene flow and dispersal to other populations, which may lead to a reduction of population abundance and increase the probability of population and meta-population extinction [28,29]. According to DDHS theory, a population at low abundance that contracts to a smaller, potentially more optimal habitat, might be more sensitive to environmental [30], demographic and genetic stochasticity [31], as well as overexploitation. Fishing in an area of high density can cause local depletion when recolonization rates are low relative to the intensity of fishing [32]. Fisheries have caused contractions in fish spatial distribution [15,33] and can reduce the spatial heterogeneity of populations, an important component of their bet-hedging or risk-spreading strategies to withstand environmental variability [34]. For example, fishes exhibit spatial variation in reproduction traits [26] and it may be only a small fraction of spawners, spawning at the appropriate time and place—which can vary annually-, that successfully contributes to each new cohort [35]. A reduction in density has potential to reduce the ability of a population to protect itself against predators [36], to locate prey [37], and to affect the probability of eggs being fertilized [38]. Given that, a reduction in (density across) spatial distribution has considerable potential to reduce recruitment and abundance and to increase population variability.

The impacts of fisheries on spatial distribution and persistence of fish populations are not well known, and potential impacts might not always be well-reflected by population abundance data [4,26]. Thus, for the purpose of conservation and resource management, it can be deemed important to have a comprehensive understanding of marine fish populations across space to ensure persistence throughout their geographic range [4].

The relationship between abundance in terms of the number of individuals and spatial distribution has been studied for several fish species, but not yet between spatial distribution and SSB, the primary currency of fisheries management and a primary indicator of the reproductive potential of fish populations [39]. Spawning biomass is generally considered a better indicator of the spawning potential than spawning number, because spawning stock biomass can be more closely related to the presence of larger, older fish, that are more successful at spawning, whereas spawning stock number tends to be more closely related to the high number of younger age classes; considered less successful reproducers [40].

Here, we examine and compare the shapes of relationships between spatial distribution indicators and SSB, to assess the functionality of fisheries management reference points based on spatial criteria. We describe the relationship between SSB and three key characteristics of spatial distribution that reflect different aspects of the distribution characteristics of fish populations: range, concentration, and density [41]. Specifically, we examine: (i) Area Occupied to measure changes in range [42]; (ii) D90% or the proportion of total survey area occupied by the top 90th percentage of population biomass (Swain and Sinclair 1994); (iii) Gini Index or evenness of the spread of biomass across an area to measure changes in concentration [42,43]; and (iv) Density Area or the proportion of tows consisting of high, medium and low biomass across the surveyed area to measure changes in density [8].

Many other indicators are available describing similar and other aspects of spatial distribution [9]. This is intended as an exploratory analysis, and we selected Area Occupied, D90%, Gini Index and Density Area, because they incorporate different information from the survey data regarding the spatial distribution of fish. Our primary objective is to determine if any of these spatial distribution metrics (SDMs) can be considered a reliable indicator for rapid changes or turning points in SSB. For the SDMs that can be considered a reliable indicator for disproportionately large SSB changes, a time series analysis is applied to detect whether these SDMs can also be considered predictive indicators that precede rapid changes in SSB in time. A time series analysis might reveal macroecological patterns where population size increases faster than range size, for example due to DDHS, such that there is a positive interspecific relationship between local density and range size. Whether the SDM leads or lags changes in SSB might also depend on whether decreases are environmental or fishing-induced. This analysis will however be restricted to the value of spatial distribution as indicators for changes in abundance, but will exclude an analysis of the causes for this particular relationships, even though the cause(s) may have affected that particular relationship, as shown by Trenkel et al. [44]. With the aforementioned analyses we explore the potential utility and development of fisheries management reference points based on spatial criteria.

## Materials and Methods

**Fig 1:**
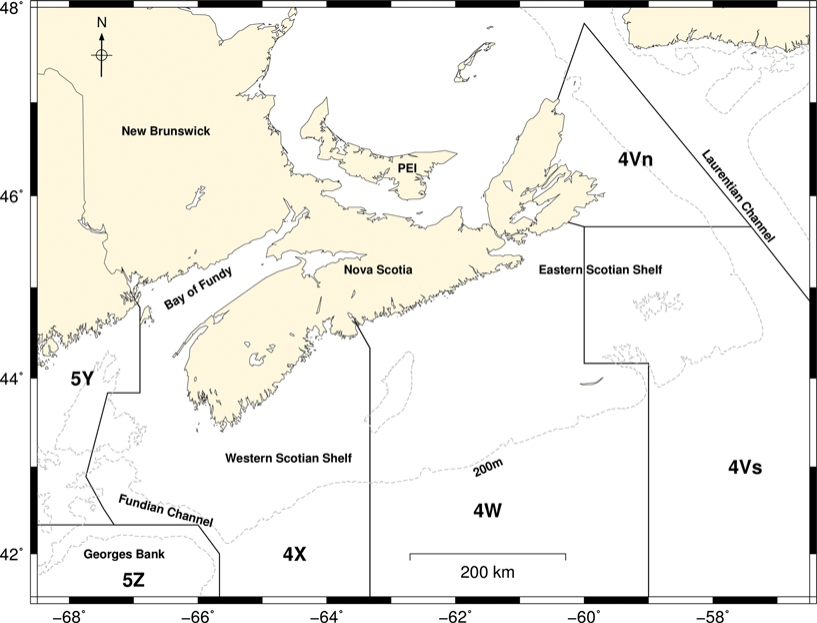
North Atlantic Fisheries Organization (NAFO) Subdivisions [46]. The western pollock NAFO subdivisions (4Xopqrs+5Yb+5Zc) are indicated in the pollock science advisory report [47].

We analyzed annual survey data of the fishery independent groundfish trawl survey of the Scotian Shelf and Bay of Fundy in Northwest Atlantic Fisheries Organization (NAFO) areas 4V, 4W, 4X, 5Y, 5Z (Fig. 1, Table 1), conducted annually since 1970 by the Department of Fisheries and Oceans (DFO). The survey follows a standardized random sampling protocol and tows follow a random sampling design within areas stratified by depth. This design enables the collection of unbiased estimates of population abundance through time [45]. The number of sets or hauls that are taken every year (within one month) is in the hundreds and differences in the survey from one year to another are considered minimal, given that spatial coverage is constant.

**Table 1:** Survey data from North Atlantic Fisheries Organization (NAFO) divisions on the Scotian Shelf.

Species	Name used	NAFO division	Years included	Maximum decline SSB
***Hippoglossoides platessoides***	American plaice	4VWX	1970–2009	64.0%
***Gadus morhua***	WSS codⁱ	4X	1980–2008	88.0%
***Gadus morhua***	ESS codⁱⁱ	4VsW	1970–2011	97.2%
***Melanogrammus aeglefinus***	Haddock	4X5Y	1970–2008	69.0%
***Hippoglossus hippoglossus***	Halibut	4VWX	1970–2009	77.1%
***Pollachius virens***	Pollock	4Xopqrs+5Yb+5Zc	1982–2008	88.5%
***Sebastes spp.***	Redfish	4X+4Wdehkl (Unit 3)	1970–2012	93.0%
***Merluccius bilinearis***	Silver hake	4VWX	1993–2012	94.2%
***Urophycis tenuis***	White hake	4X	1970–2011	94.3%
***Pseudopleuronectes americanus***	Winter flounder	4X	1970–2011	99.4%

^ⁱ^WSS = Western Scotian Shelf,

^ⁱⁱ^ESS = Eastern Scotian Shelf.

This table shows the years included in Spawning Stock Biomass (SSB) and Spatial Distribution Metric (SDM) timeseries and maximum percentage of decline compared to maximum value of SSB recorded in the respective timeseries.

We assume that the stock areas encompass a meta-population or a series of related populations and assume minimal immigration or emigration. Spatial distribution is calculated within the same NAFO area for which the abundance metric, i.e. Spawning Stock Biomass (SSB) from DFO stock assessments, has been estimated and we correct spatial distribution metrics for differences in stratum area. SSB estimates are based on DFO stock assessment models which vary per species (specified in S1 Stock Assessment).

The species we examine include American plaice (*Hippoglossoides platessoides*), cod (*Gadus morhua*), haddock (*Melanogrammus aeglefinus*), halibut (*Hippoglossus hippoglossus*), pollock (*Pollachius virens*), redfish (*Sebastes* spp.), silver hake (*Merluccius bilinearis*), white hake (*Urophycis tenuis*) and winter flounder (*Pseudopleuronectes americanus*). These species are or have been exploited commercially and have experienced large changes in SSB, increasing the likelihood of detection of a relationship between SSB and the Spatial Distribution Metric (SDM). The populations, their location, years included in each analysis, and the magnitude of SSB decline are reported in Table 1. We did not apply a size threshold to the SDM, because information regarding maturity is not available for every species.

Area Occupied (AO) is a measure of range. This is a presence and absence measure of the amount of area that is occupied. The proportion of area occupied in survey year *k*, by stock *n*, is calculated as the sum over all strata of the proportions of tows, with catch *c* in each stratum *s*, and multiplied by the area *A* of the respective stratum. This, in turn, is divided by the total area to calculate the proportion of area occupied *AO*_*k*,*n*_:

$$AO_{k,n}=\frac{\sum_{s=1}^{S_{k}}\frac{t_{k,n,s}^{c}}{t_{k,n,s}}A_{k,s}}{\sum^{\text{​}}A_{s}}$$ (1)

Where $t_{\left(k,n,s\right)}^{c}$ is the number of tows with [positive] catch (in biomass) per year, per stock, per stratum, *t*_(*k*,*n*,*s*)_ is the total number of tows per year, per stock, per stratum, and *A*_(*k*,*s*)_ is the surface area per year per stratum, Σ*A*_*s*_ is the total area [42].

D90% is a metric of concentration: the minimum area containing 90% of the survey biomass as a proportion of the total area, is calculated from information on the mean stratum-weighted biomass ($\bar{WB}$), which is calculated by taking the mean biomass $\left(\bar{B}\right)$ in survey year (*k*), per stock (*n*), per stratum (*s*), weighted by the proportion of surface area (*A*) in survey year (*k*), per stratum (*s*) and dividing that by its sum:

$${\bar{WB}}_{k,n,s}=\frac{{\bar{B}}_{k,n,s}*\frac{A_{k,s}}{\sum_{s=1}^{S_{k}}A_{k,s}}}{\sum^{\text{​}}({\bar{B}}_{k,n,s}*\frac{A_{k,s}}{\sum_{s=1}^{S_{k}}A_{k,s}})}$$ (2)

This yields${\bar{WB}}_{k,n,s}$, the weighted proportion of mean biomass per stock, per year, per stratum. These ${\bar{WB}}_{k,n,s}$ values are ordered from the highest ${\bar{WB}}_{k,n,s}$(highest biomass across area) to the lowest, until the cumulative sum of ${\bar{WB}}_{k,n,s}$equals 0.9. Then, the cumulative sum of the associated proportion of surface area is identified, and this number represents the proportion of area needed to capture 90% of the population biomass, referred to as D90%.

The Gini index is also a metric of concentration, but one that measures how evenly biomass is spread across an area. Gini index is bounded by 0 and 1 and captures the deviation from the equal distribution (Gini = 0) of the cumulative sum of ${\bar{WB}}_{k,n,s}$ versus the cumulative sum of associated proportion of surface area. The greater the Gini index value, the less evenly the spatial distribution of biomass and the greater the degree to which that biomass is spatially concentrated. To calculate the Gini coefficient, we order ${\bar{WB}}_{k,n,s}$by increasing values and calculate the cumulative proportion of the survey biomass. The Gini index per year (*k*), per species (*n*) is estimated by:

$$G_{k,n}=1-2\int_{0}^{1}L_{x}dx$$ (3)

or the area between the 1:1, or 45 degree-line (equal distribution of the biomass across area) and the Lorenz curve *L*_*x*_, which is the curve of cumulative proportion of biomass versus the cumulative proportion of area multiplied by 2. Thus, the Gini index also incorporates zero biomass per stratum, which could indicate either a real absence or the wrongful inclusion of an area that does not belong to the species domain. To reduce the probability of wrongful inclusion, we excluded those strata (from all SDMs) that never recorded an individual of the respective species in the respective stock area across the full time series.

For density, we use a method that has been applied to northern Atlantic cod. Hutchings [8] quantified temporal changes in cod densities by partitioning research survey data into low (0–100 kg), medium (100–500 kg), and high (>500 kg) biomass levels per tow and subsequently calculating the proportion of tows falling within each category. Each tow samples approximately the same surface area of ocean bottom. We partitioned our data on survey tow densities into high, medium, and low density areas, based on quantiles of the overall biomass per tow across the entire time series for each stock, to allow for comparison among stocks. To deal with the large amount of tows containing no biomass, the quantiles were calculated without zero density tows, and we added a fourth zero density area metric. Thus, the metric is comprised of four categories, with the proportion of tows falling in the following density area categories: 0 = *Zero*, 0 <*Low≤33*.*3%*, 33.3%<*Medium≤66*.*7%* and 66.7%<*High*≤100%.

Based on previous results for Newfoundland’s northern cod [8], we anticipate for the two cod stocks on the Scotian Shelf that as SSB declines, [the proportion of] medium density areas (MDAs) will decrease and low density areas (LDAs) as well as zero density areas (ZDAs) will increase. High density areas (HDAs) are expected to decline increasingly rapidly as SSB declines. Given our expectation that the relationship between SSB and HDAs will be sensitive to the level of tow density considered to be ‘high’, we explore several HDA categories, for which we vary the quantile distribution of biomass per tow by taking the highest 33.3% of the biomass per tow quantile distribution (HDA 33), highest 15% (HDA 15), highest 10% (HDA 10), highest 5% (HDA 5), and highest 2.5% (HDA 2.5). When calculating D90%, Gini index, LDA, MDA and various categories of HDA (HDA *x*), individual size limits to exclude pre-spawners were not applied, given that information regarding size at maturity is not available for every species.

To determine whether spatial distribution variation can explain SSB variation and potentially be used as an indicator, we assess the shape of the relationship between SSB and the SDM AO, D90%, Gini index, ZDAs, LDAs, MDAs and HDAs (various levels), using a non-linear least squares regression model [6,48]:

$$SSB_{k,n}=b*SDM_{k,n}^{c}.$$ (4)

In case of a negative relationship between SSB and SDM, the SDM is inverted. This inversion allows the same model to be applied and allows for a comparison of the shape of the SDM-SSB relationship among stocks:

$$SSB_{k,n}=b*{\left(1-SDM_{k,n}\right)}^{c}.$$

**Fig 2:**
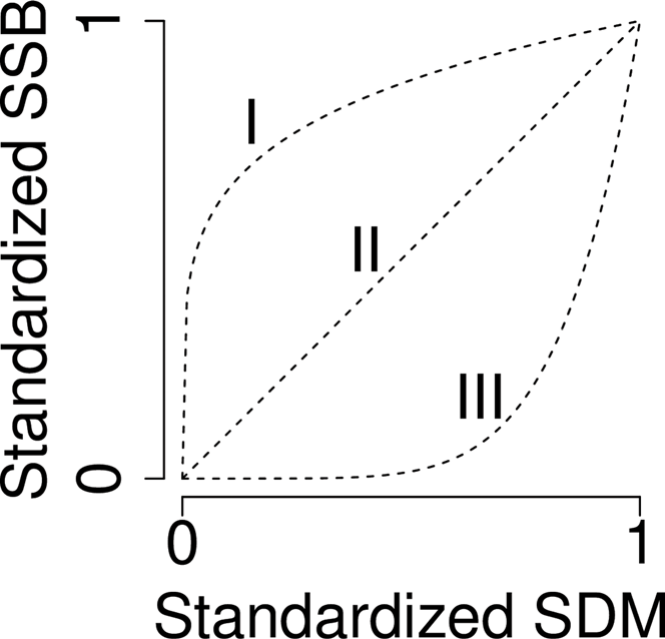
Hypothetical concave (type I), linear (type II) and convex (type III) Spatial Distribution Metric (SDM)- Spawning Stock Biomass (SSB) relationship. These relationships are generated from.SSB = b * SDM ^*c*^ An exponent or c-value >1 will generate a convex relationship, whereas a c-value < 1 will generate a concave relationship. A c-value not significantly different from 1 will generate a linear relationship.

Utilizing the model estimates, we apply a *t-*test to test whether parameter *c* is significantly different from 1. Exponent *c* determines the shape of the relationship: a type I (concave) relationship occurs when *c* < 1, a type II when *c* = 1, and type III (convex) when *c* > 1 (Fig. 2).

The SSB and the SDM are standardized by scaling each metric by its maximum recorded value, to facilitate comparison of the regression analysis among SDMs and stocks;

$$Z_{m}=\frac{m}{m_{max}}$$ (5)

Where *Z*_*m*_ is the standardized value and *m* is the metric SSB, AO, D90%, Gini index, ZDAs, LDAs, MDAs, HDA *x*, and where *m*_*max*_ is the maximum value of the respective metric. However, we refer to standardized values such as *Z*_*Ao*_, *Z*_*ssB*_ simply as AO and SSB in the text and figures. The same standardization used in model 5 is used for both the non-linear least squares regression model and the linear regression model 6.

$$SSB_{k,n}=a\pm b*SDM_{k,n}$$ (6)

In cases of both a significant linear and non-linear relationship, we selected the model with the best fit based on Akaike’s Information Criterion (AIC) [49]. Thus, the linear model is selected when *c* in the non-linear model (4) is not significantly different from 1 and/or when the linear model has the same or a lower AIC value.

It might be that in all three types of SDM-SSB relationships (concave or type I, linear or type II, convex or type III, see Fig. 2) the SDM leads or lags linear or non-linear changes in SSB. If the SDM leads changes in SSB, this could be valuable information for fisheries management because the SDM is then likely to reach a spatial value associated with a particular SSB (biomass) reference point before SSB reaches that SSB reference point. However, our time series analysis will be limited to the non-linear relationships (type I and type III). Non-linear relationships are particularly of interest when assessing the added value of spatial reference points, because they might indicate that changes in the SDM precede accelerated changes in SSB. A cross-correlation function is therefore applied to the non-linear relationships (type I and type III) to test whether the SDM leads or lags SSB by lag *h*. The cross-covariance function *γ*_*SDM*_,_*SSB*_(*h*)

$$\gamma_{SDM,SSB}\left(h\right)=cov\left(SDM_{t-h},SSB_{t}\right)=E\left[\left(SDM_{t-h}-\mu_{SDM}\right)\left(SSB_{t}-\mu_{SSB}\right)\right]$$ (7)

is used to calculate the cross-correlation function between the SDM and SSB, which yields *ϱ_SDM,SSB_*; the cross correlation between the SDM and SSB time series:

$$\varrho_{SDM,SSB}=\frac{\gamma_{SDM,SSB}\left(h\right)}{\sqrt{\gamma_{SDM}(0)*\gamma_{SSB}(0)}},\varepsilon \sim N\left(0,\sigma^{2},I\right)$$ (8)

The peak in this cross correlation defines the maximum correlation ϱ_*max*_ at lag *h* [50]. Before application of model 8, generalized least squares models are applied to transform and detrend the time series and to account for residuals that do not demonstrate approximate normal distributions, zero mean, constant variance, independence. This does not affect model 4 parameters, but yields a maximum likelihood estimate of the regression parameters that accounts for autocorrelation and autoregressive, moving average errors and therefore yields wider confidence intervals than OLS results. Thus model 8 is applied to the residuals of the detrended, auto-correlation accounted for timeseries. The data analysis is performed in R [51].

## Results

Many of the indices yielded significant associations between the Spatial Distribution Metric (SDM) and Spawning Stock Biomass (SSB) (Table 2). Populations showed considerable variability in terms of SSB sensitivity to different SDMs. For example, the SSB of haddock was spatially linked with one index; Area Occupied (AO). Alternatively, the SSB of Western Scotian Shelf (WSS) cod was significantly related to all four examined spatial distribution methods.

**Table 2:** Results of the significant Spatial Distribution Metric (SDM)-Spawning Stock Biomass (SSB) relationships for ten stocks on the Scotian Shelf.

Species	SDM	*B*			*c*				Shape
		value	S.E.	p-value	value	S.E.	p-value	p(curve)	
**Cod (ESS)**	AO	0.80	0.09	<0.001	2.49	0.53	<0.001	0.01	III
**Cod (WSS)**	AO	0.82	0.10	<0.001	2.62	0.64	<0.001	0.02	III
**Haddock**	AO	0.49	0.24	0.05	NA	NA	NA	NA	II
**Winter flounder**	AO	0.46	0.06	<0.001	1.81	0.40	<0.001	0.05	III
**American plaice**	D90%	1.00	0.33	<0.01	NA	NA	NA	NA	II*
**Cod (WSS)**	D90%	0.85	0.37	0.03	NA	NA	NA	NA	II
**White hake**	D90%	0.80	0.24	<0.01	NA	NA	NA	NA	II*
**American plaice**	Gini	0.92	0.37	0.02	NA	NA	NA	NA	II
**Cod (WSS)**	Gini	0.61	0.32	0.07	NA	NA	NA	NA	II*
**Pollock**	Gini	0.52	0.25	0.05	NA	NA	NA	NA	II
**White hake**	Gini	0.61	0.08	<0.001	2.06	0.57	<0.001	0.07	III
**American plaice**	HDA 33	0.78	0.13	<0.001	NA	NA	NA	NA	II
**Cod (ESS)**	HDA 33	0.84	0.09	<0.001	NA	NA	NA	NA	II
**Cod (WSS)**	HDA 33	0.82	0.10	<0.001	NA	NA	NA	NA	II
**Halibut**	HDA 33	0.35	0.14	0.02	NA	NA	NA	NA	II
**Pollock**	HDA 33	0.59	0.20	<0.01	NA	NA	NA	NA	II
**Redfish**	HDA 33	0.49	0.19	0.01	NA	NA	NA	NA	II
**Silver hake**	HDA 33	0.62	0.30	0.05	NA	NA	NA	NA	II
**White hake**	HDA 33	0.57	0.08	<0.001	0.64	0.21	<0.01	0.09	I
**Winter flounder**	HDA 33	0.47	0.06	<0.001	NA	NA	NA	NA	II
**Cod (ESS)**	MDA	0.91	0.24	<0.001	NA	NA	NA	NA	II
**Cod (WSS)**	MDA	0.71	0.29	0.02	NA	NA	NA	NA	II
**Winter flounder**	MDA	0.27	0.12	0.03	NA	NA	NA	NA	II
**American plaice**	LDA	0.98	0.18	<0.001	NA	NA	NA	NA	II*
**Cod (ESS)**	LDA	1.07	0.12	<0.001	7.31	1.18	<0.001	<0.001	III*
**Cod (WSS)**	LDA	0.79	0.08	<0.001	3.83	0.95	<0.001	0.01	III*
**Halibut**	LDA	3.86	1.64	0.02	NA	NA	NA	NA	II*
**Pollock**	LDA	0.81	0.11	<0.001	4.40	1.06	<0.001	<0.01	III*
**White hake**	LDA	0.96	0.41	0.02	NA	NA	NA	NA	II*
**American plaice**	ZDA	0.33	0.17	0.06	NA	NA	NA	NA	II
**Cod (ESS)**	ZDA	0.88	0.10	<0.001	3.46	0.68	<0.001	<0.001	III*
**Cod (WSS)**	ZDA	0.77	0.09	<0.001	2.81	0.76	0.001	0.02	III*
**Winter flounder**	ZDA	0.49	0.06	<0.001	2.08	0.45	<0.001	0.02	III*

The relationships are defined as a linear model *SSB* = *a±b* * *SDM* (listing NAs for c), or as a non-linear model

*SSB* = *b* * *SDM* ^*c*^, where p(curve) represents the p-value for significant deviation of the exponent value from 1. In the last column, the * symbolizes an inverted SDM, thus representing a negative relationship between the SDM and SSB.

**Fig 3:**
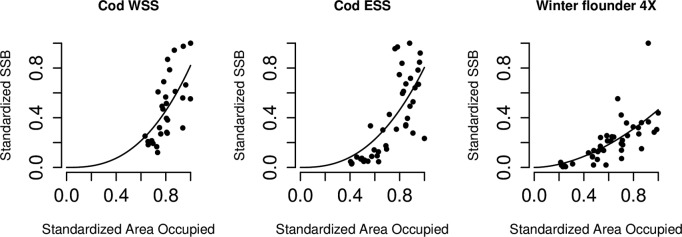
Significant convex relationships between Area Occupied (AO) and Spawning Stock Biomass (SSB) for Western Scotian Shelf (WSS) cod, Eastern Scotian Shelf (ESS) cod and between AO and Spawning Stock Number (SSN) for winter flounder 4X, according to *SSB* = b * *AO* ^*c*^.

There was a significant relationship between SSB and AO for four stocks. The relationship was linear for haddock but convex for cod and winter flounder (Fig. 3). The convex relationship for WSS cod means that a decline to 0.2 SSB or to 20% of its maximum SSB (a loss of 80% of SSB) is associated with a relatively much smaller reduction in maximum area occupied (58%, or a loss of 42% of AO). WSS and Eastern Scotian Shelf (ESS) cod showed a positive linear relationship between D90% and SSB; an increase in SSB is associated with a constant expansion of area across which 90% of the biomass is found. For American plaice and white hake, D90% showed a negative linear relationship with SSB; i.e. with an increase in SSB, there is a decrease in area across which 90% of the biomass is concentrated. D90% was not significantly related to the SSB of any of the other assessed stocks.

The relationship between SSB and the Gini index was negative and linear for WSS cod, but positive and linear for American plaice and pollock, and positive and convex for white hake. According to these relationships, as WSS cod SSB increases, the population becomes more evenly distributed, whereas the American plaice, pollock and white hake populations become less evenly distributed as SSB increases. According to its convex shape, white hake experiences a relatively faster reduction of even spread as SSB increases.

**Fig 4:**
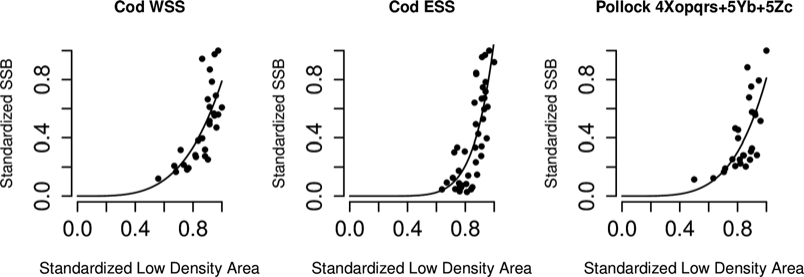
Significant convex Low Density Area (LDA)- Spawning Stock Biomass (SSB) relationships for Western Scotian Shelf (WSS) and Eastern Scotian Shelf (ESS) cod and pollock 4Xopqrs+5Yb+5Zc according to *SSB* = *b* * *LDA* ^*c*^. The LDAs are inverted, i.e. an increase on the x-axis represents a decrease in the proportion of LDAs.

Relationships between the tow-density indices and SSB varied with the index examined and varied by stock. For nine out of ten populations, High Density Area (HDA) 33 had a relationship with SSB, of which eight positive and linear and one positive and concave. Three out of ten stocks exhibited a linear relationship between Medium Density Area (MDA) and SSB, while six demonstrated a significant negative relationship between Low Density Area (LDA) and SSB, of which three linear and three convex, shown in Fig. 4. For four stocks, a significant relationship exists between SSB and Zero Density Area (ZDA), of which three are negative and convex and one (American plaice) positive and linear. This indicates that, for American plaice, SSB increases with an increase in the frequency of encountering ZDAs, a result consistent with the patterns of increased concentration seen in the relationships of D90% and the Gini index with SSB for this species.

**Fig 5:**
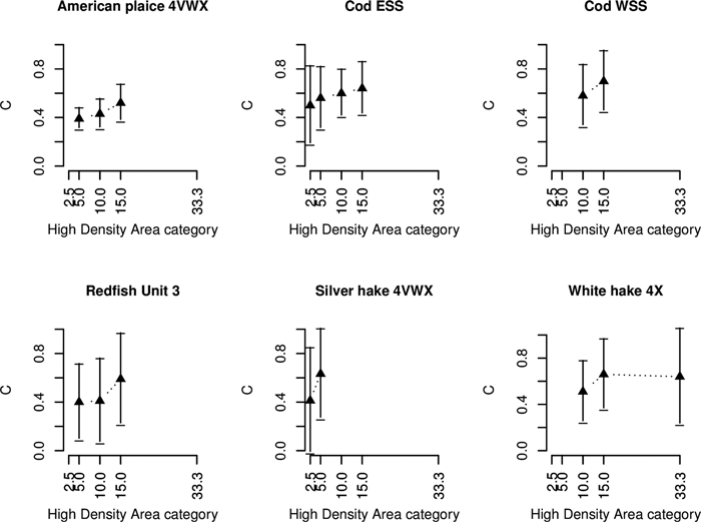
Exponent c values with 95% confidence interval for different categories of High Density Area (HDA) for six stocks on the Scotian Shelf from significant concave relationships according to *SSB* = *b* * *HDA* ^*c*^.

SSB was significantly related to several high-density area categories in nine out of ten populations (Table 3). With the exception of a convex relationship between SSB and HDA 15 (proportion of tows falling in the the highest 15% of the biomass per tow quantile distribution) for winter flounder, all relationships are positive and either linear or concave. For various high density area categories, there is good evidence for a non-linear concave relationship with SSB, demonstrated by the exponent *c* values staying below 1 (Fig. 5). The concave relationship appears robust for ESS cod, as HDA 15, 10, 5, 2.5 all demonstrate a significant, positive and concave relationship with SSB. The same significant concave relationship is found between white hake HDA 33, 15, 10 and SSB; Redfish and American plaice HDA 15, 10, 5 and SSB; WSS cod HDA 15 and 10 and SSB; and silver hake HDA 5 and 2.5 and SSB (Fig. 5). Pollock on the other hand, expressed non-significant concave relationships between HDA and SSB categories, with exponent values of 1 lying within its 95% confidence interval and with linear models proving a more appropriate fit. This is also the case for winter flounder, except where the exponent value for HDA15 is significantly larger than 1, making the relationship between SSB and HDA 15 convex.

**Table 3:** Results of the significant relationships between various levels of High Density Area (HDA) and Spawning Stock Biomass (SSB).

Species	SDM	b			c				Shape
		value	S.E.	p-value	value	S.E.	p-value	p(curve)	
**American plaice**	HDA 15	0.91	0.05	<0.001	0.52	0.08	<0.001	<0.001	I
**Cod (ESS)**	HDA 15	0.92	0.09	<0.001	0.64	0.11	<0.001	<0.01	I
**Cod (WSS)**	HDA 15	0.91	0.09	<0.001	0.70	0.13	<0.001	0.03	I
**Halibut**	HDA 15	0.34	0.11	<0.01	NA	NA	NA	NA	II
**Pollock**	HDA 15	0.49	0.19	0.01	NA	NA	NA	NA	II
**Redfish**	HDA 15	0.60	0.09	<0.001	0.59	0.19	<0.01	0.04	I
**Silver hake**	HDA 15	0.75	0.27	0.01	NA	NA	NA	NA	II
**White hake**	HDA 15	0.65	0.08	<0.001	0.66	0.15	<0.001	0.03	I
**Winter flounder**	HDA 15	0.83	0.08	<0.001	1.30	0.14	<0.001	0.04	III
**American plaice**	HDA 10	0.87	0.05	<0.001	0.43	0.06	<0.001	<0.001	I
**Cod (ESS)**	HDA 10	0.91	0.08	<0.001	0.60	0.10	<0.001	<0.001	I
**Cod (WSS)**	HDA 10	0.79	0.07	<0.001	0.58	0.13	<0.001	<0.01	I
**Halibut**	HDA 10	0.32	0.12	<0.01	NA	NA	NA	NA	II
**Pollock**	HDA 10	0.54	0.16	<0.01	NA	NA	NA	NA	II
**Redfish**	HDA 10	0.54	0.09	<0.001	0.41	0.18	0.02	<0.01	I
**Silver hake**	HDA 10	0.72	0.21	<0.01	NA	NA	NA	NA	II
**White hake**	HDA 10	0.65	0.08	<0.001	0.51	0.14	<0.001	<0.001	I
**Winter flounder**	HDA 10	0.57	0.07	<0.001	NA	NA	NA	NA	II
**American plaice**	HDA 5	0.86	0.04	<0.001	0.39	0.05	<0.001	<0.001	I
**Cod (ESS)**	HDA 5	0.90	0.12	<0.001	0.56	0.13	<0.001	<0.01	I
**Cod (WSS)**	HDA 5	0.51	0.18	0.01	NA	NA	NA	NA	II
**Halibut**	HDA 5	0.24	0.12	0.04	NA	NA	NA	NA	II
**Pollock**	HDA 5	0.49	0.14	<0.01	NA	NA	NA	NA	II
**Redfish**	HDA 5	0.62	0.11	<0.001	0.40	0.16	0.02	0.001	I
**Silver hake**	HDA 5	0.89	0.15	<0.001	0.63	0.19	<0.01	0.07	I
**White hake**	HDA 5	0.70	0.10	<0.001	NA	NA	NA	NA	II
**Winter flounder**	HDA 5	0.68	0.09	<0.001	NA	NA	NA	NA	II
**American plaice**	HDA 2.5	0.56	0.07	<0.001	NA	NA	NA	NA	II
**Cod (ESS)**	HDA 2.5	0.94	0.19	<0.001	0.50	0.16	<0.01	<0.01	I
**Cod (WSS)**	HDA 2.5	0.39	0.18	0.04	NA	NA	NA	NA	II
**Redfish**	HDA 2.5	0.45	0.13	<0.001	NA	NA	NA	NA	II
**Silver hake**	HDA 2.5	0.78	0.17	<0.001	0.41	0.22	0.08	0.02	I
**White hake**	HDA 2.5	0.78	0.09	<0.001	NA	NA	NA	NA	II
**Winter flounder**	HDA 2.5	0.73	0.09	<0.001	NA	NA	NA	NA	II

The relationship is defined as a linear model *SSB* = *a±b* * *SDM* (listing NAs for c), or as a non-linear model

*SSB* = *b* * *SDM* ^*c*^, where p(curve) represents the p-value for significant deviation of the exponent value from 1.

**Fig 6:**
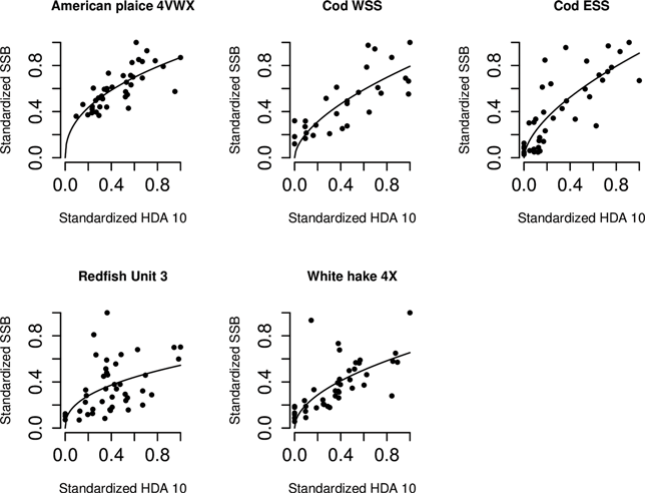
Significant positive and concave High Density Area (HDA) 10-Spawning Stock Biomass (SSB) relationships according to *SSB* = *b* * (*HDA*10)^*c*^.

For most stocks, the spatial distribution methods HDA 5 and HDA 2.5 contained a large number of zeros. The HDA 15 and HDA 10 categories generally contained few zeros, and SSB appears most sensitive to changes in HDA15 and HDA 10, with a concave shape for five stocks in our analysis (Fig. 6).

Of the significant concave and convex relationships, there are a few in which the SDM precedes changes in SSB, indicated by the negative lag *h* in Table 4. All other stocks with a concave or convex SDM-SSB relationship not listed in Table 4 were found to be maximally correlated at a zero lag.

**Table 4:** Results of the time series analyses of concave (type I) and convex (type III) Spatial Distribution Metric (SDM)- Spawning Stock Biomass (SSB) relationships wherein SDM precedes changes in SSB, indicated by negative lag h.

Species	SDM	Maximum significant Cross Correlation (ϱ_*max*_)		Type SDM-SSB relation-ship	SDM time series residuals via	SSB time series residuals via
		*ϱ_SDM,SSB_*	Lag (*h*) in year(s)			
**Cod (ESS)**	AO	0.671	-1	III	GLS-AR1	Log GLS-AR1
**Cod (WSS)**	AO	0.442	-1	III	OLS	Log GLS-AR1
**Winter flounder**	AO	0.378	-2	III	OLS	Log GLS-AR1
**Cod (ESS)**	LDA	0.520	-3	III*	GLS-AR1	Log GLS-AR1
**Cod (ESS)**	ZDA	0.640	-1	III*	GLS-AR1	Log GLS-AR1
**Winter flounder**	ZDA	0.313	-2	III*	OLS	Log GLS-AR1
**American plaice**	HDA 15	-0.412	-3	I	OLS	GLS-AR1
**Cod (ESS)**	HDA 15	0.719	-1	I	GLS-AR1	Log GLS-AR1
**Cod (WSS)**	HDA 15	0.521	-1	I	OLS	Log GLS-AR1
**American plaice**	HDA 10	0.404	-6	I	OLS	GLS-AR1
**Cod (ESS)**	HDA 10	0.690	-1	I	GLS-AR1	Log GLS-AR1
**Cod (WSS)**	HDA 10	0.469	-1	I	OLS	Log GLS-AR1
**Cod (ESS)**	HDA 5	0.585	-2	I	GLS-ARMA (2,1)	Log GLS-AR1
**Cod (ESS)**	HDA 2.5	0.515	-1	I	GLS-AR1	Log GLS-AR1

The time series residuals were obtained using OLS: ordinary least squares regression, GLS-AR1: generalized least squares regression with an auto-correlation or auto-regressive process of order 1, GLS-ARMA (p,q): generalized least squares regression with a p order auto-regressive and q order moving average process, Log: indicates that the SSB time series was log-transformed before applying regression analysis.

*Indicates the SDM is inverted. A negative *ϱ*_*SDM*_,_S*SB*_ indicates a negative relationship between SDM and SSB.

**Fig 7:**
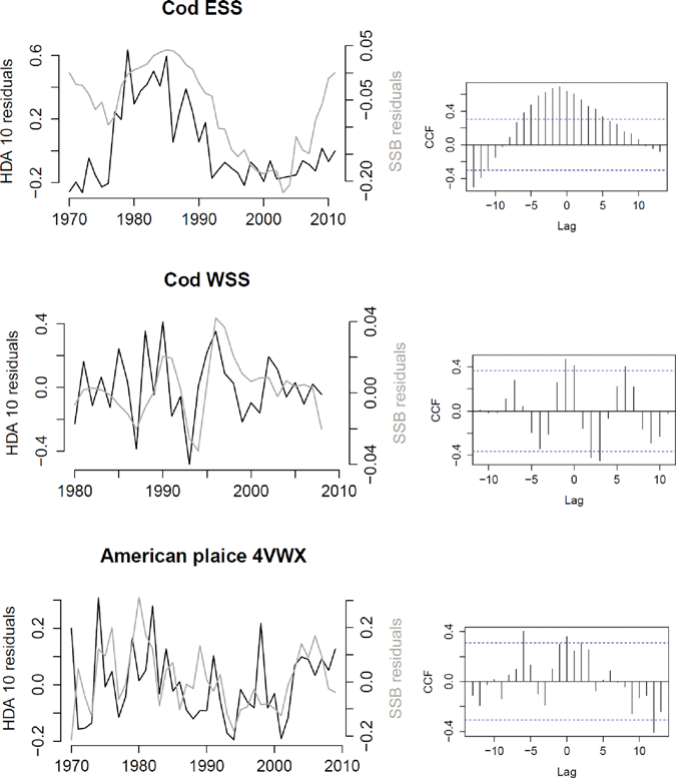
High Density Area (HDA) 10-Spawning Stock Biomass (SSB) time series (left) and associated cross correlation functions (right). These show maximum cross correlation between HDA 10 and SSB at a lag h = -1 for Western Scotian Shelf (WSS) cod and Eastern Scotian Shelf (ESS) cod and h = -6 for American plaice (See Table 4 for the models used to create the time series residuals).

For cod, HDAs precede declines in SSB with a one year time lag, except ESS cod HDA 5, which leads SSB with a two year time lag. The time series and cross correlation between ESS and WSS cod HDA 10 and SSB are depicted in Fig. 7. The cross correlation function demonstrates that maximum cross correlation occurs when ESS cod HDA 10 leads changes in SSB with a one year time lag. Cross correlation is also high (although lower than the maximum cross correlation at *h* = -1) at other time lags, including positive lags. The time series show that it is mainly the rapid declines (not increases) in SSB that are preceded by HDA 10 declines. For WSS cod, the large SSB increase leading up to 1990 and 1996 and decline afterwards were preceded by an increase and decrease in HDA 10. For American plaice the cross-correlation between HDA 10 and SSB is maximum at a lag of *h* = -6, although a cross correlation at a lag of *h* = 0 is presumably more likely.

**Fig 8:**
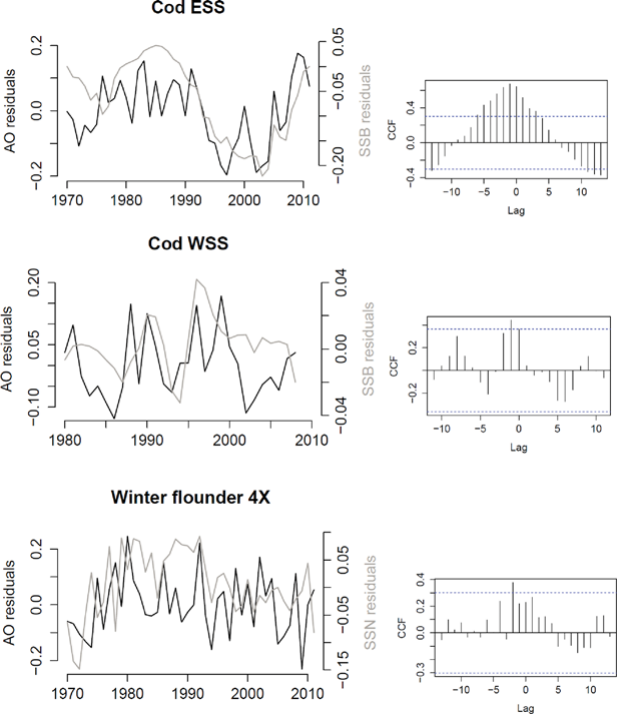
Area Occupied (AO) and Spawning Stock Biomass (SSB) time series (left) and associated cross correlation functions (right). These show maximum cross correlation between AO and SSB at a lag h = -1 for Western Scotian Shelf (WSS) cod and Eastern Scotian Shelf (ESS) cod and h = -2 for winter flounder (See Table 4 for the models used to create the time series residuals).

AO precedes changes in SSB for ESS and WSS cod and winter flounder (Table 4) and the time series and cross correlation functions are depicted in Fig. 8. For ESS cod, AO increases before SSB, whereas the SSB decline appears to happen more simultaneously with AO declines. For WSS cod the 1990 and 1996 SSB peak of WSS cod are visibly preceded by an increase in AO. For winter flounder the AO peaks appear to approximately align with SSN peaks when AO leads by two years, but not very clearly across the entire time series and this is reflected by a relatively low *ϱ*_*A0*_,_*SSB*_ value.

## Discussion

We find that the reproductive component of marine fish populations is related to their spatial distribution. Among the spatial indices that have been used for marine fishes in the past, some are more sensitive to and better predictors of changes in Spawning Stock Biomass (SSB) than others. It is these indices that are likely to be of greatest utility when identifying distributional targets for population recovery and establishing spatially informative limit reference points to prevent population collapse.

Of the Spatial Distribution Metrics (SDMs) examined for ten groundfish populations, the High Density Area (HDA) metric was found to be the SDM most frequently (significantly) related to SSB. HDAs generate type I or concave relationships with SSB. In a type I relationship, SDMs decline faster than SSB, until HDAs have declined to a point where each additional HDA decline is associated with a relatively larger SSB decline. For cod, declines in HDA also appear to precede rapid declines in SSB in time. This makes HDAs a potential indicator for forecasting imminent SSB declines, and supports further development of HDAs as a primary correlate of SSB for fisheries management purposes.

The other SDMs have either a linear (type II) or convex relationship (type III) with SSB. For example, a type II relationship is evident between SSB and D90% for cod, a pattern consistent with the results reported for cod abundance and D90% by Swain and Sinclair [14] and for abundance and D95% by Blanchard et al. [52]. SDMs that reflect a type II with SSB indicate no differential loss of range, concentration or density. These SDMs might provide an independent measure that could help decrease sampling variance and prove useful as a proxy for SSB when resources or data do not permit an annual assessment of SSB. In those cases where SDMs precede changes in SSB in time, they might also be useful as an indicator for forecasting SSB changes. However, the SSB changes would be proportional to SDM changes and the SDM would not be able to signal accelerated declines or increases in SSB. When SSB data are available and when the SDM has no ability to predict changes in SSB, the SDMs that are linearly related with SSB will not provide additional information on SSB status, and are unlikely to be informative indicators of population collapse or (fast) recovery.

Type III relationships are mainly found between Area Occupied (AO) and SSB (positive relationship) and between Low Density Areas (LDAs) and Zero Density Areas (ZDAs) and SSB (negative relationship). For populations that follow a type III or convex relationship, the decline in SDM values occurs relatively slower than the decline in SSB values. This means that a stock can experience large SSB declines, while SDM values remain relatively high. For example, the convex relationship between Western Scotian Shelf (WSS) cod AO and SSB demonstrates that an AO reduction from maximum to slightly lower levels is associated with a relatively large drop in SSB, implying proportionally greater decreases in high density areas versus low density area, which may indicate overfishing of highly dense areas. This relationship might emerge from SSB ‘spreading thin’, whereby a population’s SSB drains while the population still manages to retain a relatively large range and numerous lower density areas. A decrease of ZDAs and LDAs in favour of higher density areas could potentially indicate an increased probability of migration among patches that are occupied.

Conversely, a relatively large SSB increase in a type III relationship does not occur until these SDMs reach relatively high levels. For example with a convex relationship between SSB and AO, it appears a minimum level of area needs to be occupied, before associated high values of SSB can be observed. This could indicate a need for recolonization of an area [53]. The type III relationship between AO and SSB (cod and winter flounder) is consistent with previous research reporting declines in abundance of greater magnitude than concomitant changes in area of occupancy [42,53,54]. Increases in AO also appear to precede rapid increases in SSB for cod and winter flounder. SDMs that generate type III relationships with SSB may prove useful as indicators, with regard to a spatial distribution level and a level of recolonization that a stock needs to attain before it might achieve recovery of SSB values to relatively high levels. In this way, a particular level of recolonization could serve as a minimum criterion in a recovery plan.

The ability of HDAs in type I relationships to indicate a level below which relatively large SSB declines occur—and in the case of cod the ability of HDAs to signal upcoming large SSB declines in time—could be the result of a number of ecological processes. According to Density Dependent Habitat Selection (DDHS), realized habitat suitability will vary at different levels of abundance [55]. If ideal free distribution holds and DDHS is at play, population density will map habitat suitability and accordingly HDAs will be indicative of productive habitat. A differential loss of HDAs, possibly a result of the overexploitation of HDAs that would have a higher catch-per-unit-effort [56], may thus reflect a loss of density across highly productive areas. Losing highly productive areas could negatively affect reproduction and survival and consequently reduce SSB. The perceived ability of HDAs to signal a decline in SSB—particularly in SSB of harvested populations—is consistent with results for northern cod, for which HDAs were shown to be a leading indicator of the collapse [8]. These northern cod HDAs consisted of the highest 2–3% of the biomass per tow quantile distribution. In addition to Scotian Shelf cod, it was found that American plaice, redfish and white hake all demonstrate a relatively faster decline in both HDA 10 and HDA 15 than in SSB, until a threshold is reached below which each additional loss of a HDA is associated with relatively larger SSB declines.

With the type I relationship, we observe that a relatively rapid SSB recovery from low levels is associated with a relatively small increase in HDAs. The basin model—founded on DDHS—suggests that density is highest where habitat is most suitable, such that any particular location is thought to reflect a density dependent reduction of realized suitability [13]. HDAs can thus be thought of as a reflection of areas of high resource value and high realized suitability, and this may be the reason we observe high SSB levels with relatively few additional HDAs. At a certain point, the suitability of these optimal HDA habitats will decline and the population will start to expand into other areas, eventually creating more HDAs. This process may be supported by re-colonization of areas through increases in area of occupancy and reductions in ZDAs and LDAs, enabling an increase of Medium Density Areas (MDAs) and eventually HDAs; trends we see for example in Eastern Scotian Shelf (ESS) cod. In addition to the possibility that HDAs represent highly realized suitable habitat, they also reflect high density aggregations. The densities associated with a high SSB might reflect an aggregation behaviour that has maximized fitness, and losing these HDAs might reduce fitness advantages that come with having more individuals per unit area. These advantages can include protection from predators [36], increased probability of locating prey [37], and an increased probability of eggs being fertilized [38]. Thus, when beneficial high density aggregations associated with high SSB are reduced, decreased ability to spawn, feed and escape predators may lead to a reduction in individual fitness (Allee effect) and consequently the SSB of that fish population.

Relative to the other SDMs, HDAs generated the highest number of significant relationships with SSB across the examined fish populations. This might be because HDAs contain information about actual biomass per tow per unit of area, whereas presence and absence data (AO), or taking the mean biomass (D90% and Gini index), may not be always be sensitive enough to pick up changes in SSB. Also, considering that no size threshold could be applied and that consequently SDMs measured both spawners and pre-spawners, the SDMs may have been overestimated in years when biomass consisted mostly of pre-spawners, which might also affect the shape of the SDM-SSB relationship. An overestimation of AO and LDA might explain a relatively steep convex relationship with SSB for cod and pollock. Since HDA measures the areas of the highest density and these areas are likely to contain actual spawners, this could lead to HDA being a better indicator of SSB compared to the other SDMs.

The models used to estimate Scotian Shelf SSB are based on models considering survey biomass per tow information to a varying degree (see S1 Stock Assessment). If a model to estimate SSB is highly influenced by biomass per tow data, it would likely be strongly related to the density area metrics, and it could be argued that a relationship might stem from a lack of independence between the two variables. Indeed, SSB from stock assessments is estimated from an extrapolation of number or biomass per tow information. However, SSB is also likely to deviate from biomass per tow information, considering that stock assessments also include many other types of information regarding (and uncertainty and error associated with) total catches, maturity, sex-ratio and natural mortality [6,57,58]. Thus, biomass per tow is incorporated into the final SSB estimate to varying degrees. In addition, SSB is often based on an *average* biomass per tow, which could lead to important information being lost [8].

For this reason, the average biomass per tow was not used, which can be expected to be linearly related to SSB. Rather, biomass was divided between low, medium and high density to determine whether these could be considered sensitive indicators of rapid SSB changes, i.e. which have non-linear relationships with SSB. Generally concave relationships between SSB and HDAs were found and linear relationships between SSB and MDAs. Thus, monitoring values closer to average biomass per tow and leaving out spatial information regarding important HDAs renders the indicator less sensitive to changes in SSB.

Similar to the relationship between abundance and the catchability coefficient [59], density dependent processes may underlie the relationship between SSB and HDA. Catchability or q is the proportion of the stock caught per unit effort, and it is affected by availability of the fish stock to the gear (how many fish are in the area at the time of the haul), and catching efficiency (how many fish can be retained in the net) [60]. When abundance declines, a larger proportion of total abundance may become available to commercial fishing gear because of DDHS, which can increase catchability [8]. This can cause fishery catch-per-unit-effort (CPUE) to be hyperstable, i.e. CPUE stays high despite a decrease of abundance [48,59,61]. The concave relationships between HDA and SSB might indicate that a larger proportional loss of total biomass occurs with each additional loss of HDA, arguably due to DDHS. This process might also be explained by mathematics, since a higher proportion of the total biomass is likely located within the HDA as total biomass declines. This is however not a (mathematical) rule, considering that a range of values of the biomass per tow distribution are incorporated into HDA *x* (Table 3). The DDHS mechanism that could explain the shape of the relationships between catchability q and abundance and between SSB and HDA *x* may be the same, however, q and HDA measure something different. When q is high, there is a higher mean proportion of total biomass caught per unit effort, but the division between areas of low, medium and high abundance is not taken into account and a pattern of disappearing HDAs may be left undetected. When HDA is high, there are relatively many areas containing a high biomass, since HDA measures the proportion of total tows that contain only high densities. Therefore, HDA could arguably be considered a more direct and sensitive spatial measure of the aggregating behaviour of fish.

Reasons for not observing a relationship between SSB and a SDM might be attributed to the magnitude of change being insufficient. When spatial distribution falls to sufficiently low levels a change in SSB will emerge, justifying the forcing of model 5 through the origin (a conventional practice in fisheries science applied for example to stock-recruitment relationships; [57]). However, populations may not show this until extremely low levels. Not seeing changes in range when SSB is increasing could also be attributed to concentration providing benefits, while preferred habitat is limited or unsaturated, or both [32,62]. This might provide an explanation for the negative relationship between D90% and SSB, and between ZDAs and SSB for American plaice, whereby an increase in SSB is associated with an increase in ZDAs and a decrease in the area across which 90% of the biomass is concentrated.

SDMs showing both type I and type III relationships with SSB might be useful metrics of population collapse and recovery. Both yield information about quantities of range, concentration and density associated with productive levels of SSB and potential thresholds associated with collapse or recovery. Thus the spatial distribution of SSB can be utilized at low and at high levels and monitor stocks in their path towards recovery and particular levels can be set as minimum criteria in recovery plans. Of the SDMs that demonstrate a type I or type III relationship with SSB, HDAs could most often signal changes in SSB (six out of ten stocks). AO, LDAs and ZDAs each signal disproportional changes in SSB for three out of ten stocks. HDA appears to be a sensitive indicator, especially at lower SSB levels, whereas e.g. AO and LDAs and ZDAs display a range of values at lower SSB. SDMs that have a type III relationship with SSB would therefore not be appropriate for indicating disproportionately large SSB declines, but HDAs would be appropriate.

The relationships between SSB and the various SDMs might not necessarily represent causal relationships, nor do they necessarily indicate that DDHS is at play. It may be that SSB and the SDM co-vary with an un-parameterized, density-independent variable [17]. It may also be that the SDM is reflecting variations of the stock trait diversity or age structure. Irrespective of the causal mechanism or drivers, it appears there may be merit in further exploring how HDAs could serve as a spatial distribution indicator for SSB and how developing reference points based on spatial criteria could contribute to and complement currently used biomass reference points in fisheries management.

Limit and target reference points for fisheries could include indicators for spatial distribution and set targets for the recovery of reproductive potential throughout the geographic region of these stocks. Fisheries managers could set a limit reference point for fishing pressure to avoid the threshold of encountering HDAs associated with large SSB declines. Target reference points can help to minimize fishing pressure until HDA levels are reached that correspond to healthy SSB levels. The expansion of HDAs will most likely be preceded by recolonization of empty areas to more densely populated areas. The increase in occupied area and the shift from lower to higher density areas can be monitored during recovery and used to estimate expected recovery trajectories. As such, integrating relevant spatial distribution information into fisheries management and recovery plans will help reduce the risks and uncertainties associated with a plan based solely on the SSB criterion.

## Supporting Information

S1 Dataset Dataset Stock assessment data per species. Dryad repository: doi:10.5061/dryad.cp8pj
(CSV)

S2 Dataset Dataset SSB and source data. Dryad repository: doi:10.5061/dryad.cp8pj
(CSV)

S1 Stock Assessment Stock assessment model information. Dryad repository: doi:10.5061/dryad.cp8pj
(DOCX)
