# Supplementary material for: The Potential for Spatial Distribution Indices to Signal Thresholds in Marine Fish Biomass
Source: PLoS One. 2015 Mar 19;10(3):e0120500. doi: 10.1371/journal.pone.0120500 (PMC4366403; doi:10.1371/journal.pone.0120500)
Supplement: S1 Stock Assessment — Dryad repository: doi:10.5061/dryad.cp8pj (DOCX) [file pone.0120500.s003.docx]

| **Stock** | **SSB model** | **Source** |
| --- | --- | --- |
| American plaice | SSB is based on a stage based model and a numbers-to-tons equivalence of q-adjusted survey numbers at length | [63] |
| Cod | A VPA model is used to estimate SSB. This model uses survey and fishery catch at age, as well as an assumed natural mortality to reconstruct the numbers at age. The SSB is estimated from the total numbers at age, using mean weight and maturity ogive (proportion of the population that is mature) at age by year, as well as the fraction of mortality before spawning | Bedford Institute of Oceanography, Dept. Fisheries & Oceans, Don.Clark@dfo-mpo.gc.ca, personal communication.  4VSW data [64]  4X data: ages ≥ 3 years [65] |
| Haddock | Spawning stock biomass is estimated from an age-based Sequential Population Analysis (SPA, also known as a virtual population analysis) using the ADAPT framework, which has commercial catch numbers at age, research vessel survey stratified mean numbers at age per tow and ITQ survey total numbers at age as input and the model estimates fishing mortality population numbers and biomass. SSB is calculated as population biomass at ages 4 and older. | [66]  [67]  [68] |
| Halibut | VPA model using Research vessel survey and industry survey: A length-based, age-structured model was fitted to the length compositions in the catch and to the catch rate and length composition of halibut caught in the Scotia-Fundy groundfish Research Vessel survey (1970-2009) and halibut survey (1998-2009). | [69] |
| Pollock | A VPA model is used to estimate population abundance and fishing mortality and a proxy for SSB is the age 4+ biomass, calculated using population numbers and population (survey) average weight at age. | DFO Maritimes Science, St. Andrews, [Heath.Stone@dfo-mpo.gc.ca](mailto:Heath.Stone@dfo-mpo.gc.ca), personal communication |
| Redfish | The Scotian Shelf summer survey from 1970-2011 was used as the biomass index series. The index is in Western IIa swept area biomass equivalents. Estimated from catch and survey data, using individuals >23 cm to estimate SSB. | DFO Maritimes Science, Bedford Institute of Oceanography [Peter.Comeau@dfo-mpo.gc.ca](mailto:Peter.Comeau@dfo-mpo.gc.ca), personal communication.  [70] |
| Silver hake | A logistic biomass dynamic model was fitted to commercial fishery catches and the summer RV survey biomass index from 1993-2012 to estimate trends in population biomass and fishing mortality rates, and provide biomass projections and harvest scenarios for 2013 (Cook 2013). Model fits to the q-corrected summer RV survey biomass index captured the overall trends, as there was general coherence between the time series of survey data and model estimates. | [71]  [72] |
| White hake | SSB direct from Jim Simon using the number of fish caught with a growth parameter based on length and weight, then summing individuals of > 45 cm to estimate SSB. | DFO Maritimes Science, Bedford Institute of Oceanography [jim.simon@dfo-mpo.gc.ca](mailto:jim.simon@dfo-mpo.gc.ca),  personal communication. |
| Winter flounder | Given that SSB data are not available for winter flounder (*Pseudopleuronectes americanus*); we use Spawning Stock Number (SSN), which can be considered proportional to SSB. We obtained the SSN or Mature Female Numbers from Dr. Fowler, who informed us that Winter flounder has never been assessed with a population model (thus is derived from survey data). The model used was a derivation of a stage-based model applied to American plaice. A major consideration for winter flounder that the draft model does not address is a likely mismatch between survey and species distribution. For all the caveats, the survey is likely a pretty good source of SSN estimates, as adults are much better tracked than younger fish. Winter flounder can be converted to SSB, as Fowler did not find any evidence of changes in growth over time. But SSB has not been estimated, and therefore we used numbers, as proportionality can be assumed. | DFO Maritimes Science, Bedford Institute of Oceanography [Mark.Fowler@dfo-mpo.gc.ca](mailto:Mark.Fowler@dfo-mpo.gc.ca), personal communication |

63. DFO. Investigating reference points for American plaice on the Scotian Shelf (4VWX). DFO Can Sci Advis Sec Sci Advis Rep. 2012;2012/030.

64. Swain DP, Mohn RK. Forage fish and the factors governing recovery of Atlantic cod (*Gadus morhua*) on the eastern Scotian Shelf. Can J Fish Aquat Sci. 2012;69:997–1001.

65. Clark DS, Emberley J. Assessment of Cod in Division 4X in 2008. DFO Can Sci Advis Sec Res Doc. 2010;2009/018.

66. Mohn RK, Trzcinski MK, Black GAP, Armsworthy S, Young GA, Comeau PA, et al. Assessment of the status of division 4X5Y Haddock in 2009. DFO Can Sci Advis Sec Res Doc. 2010;2010/085.

67. DFO. Assessment of the status of 4X5Y Haddock in 2011. Can Sci Advis Sec Sci Advis Rep. 2012;2012/023.

68. Hurley, P.C.F., Black GAP, Comeau PA, Mohn RK. Assessment of 4X Haddock in 1998 and the First Half of 1999. DFO Can Stock Assess Sec Res Doc. 1999;1999/147.

69. Trzcinski MK, Armsworthy SL, Wilson S, Mohn RK, Campana SE. Framework for the assessment of the Scotian Shelf and Southern Grand Banks Atlantic halibut stock. DFO Can Sci Advis Sec Res Doc. 2011;2011/002.

70. DFO. Reference points for redfish (*Sebastes mentella* and *Sebastes fasciatus*) in the northwest Atlantic. DFO Can Sci Advis Sec Sci Advis Rep. 2012;2012/004.

71. DFO. 2012 Assessment of 4VWX Silver Hake. Can Sci Advis Sec Sci Advis Rep. 2013;2013/018.

72. Cook AM. Bayesian state space surplus production model for 4VWX silver hake. DFO Can Sci

Advis Sec Res Doc. 2013;2013/009.
